# Serum-Sphingosine-1-Phosphate Concentrations Are Inversely Associated with Atherosclerotic Diseases in Humans

**DOI:** 10.1371/journal.pone.0168302

**Published:** 2016-12-14

**Authors:** Irina Soltau, Eileen Mudersbach, Markus Geissen, Edzard Schwedhelm, Martin S. Winkler, Maria Geffken, Sven Peine, Gerhard Schoen, E. Sebastian Debus, Axel Larena-Avellaneda, Guenter Daum

**Affiliations:** 1 Clinic and Polyclinic for Vascular Medicine, University Heart Center, University Medical Center Hamburg-Eppendorf, Hamburg, Germany; 2 Institute of Clinical Pharmacology and Toxicology, University Medical Center Hamburg-Eppendorf, Hamburg, Germany; 3 Center for Anesthesiology and Intensive Care Medicine, University Medical Center Hamburg-Eppendorf, Hamburg, Germany; 4 Institute of Transfusion Medicine, University Medical Center Hamburg-Eppendorf, Hamburg, Germany; 5 Institute for Medical Biometry and Epidemiology, University Medical Center Hamburg-Eppendorf, Hamburg, Germany; Nagoya University, JAPAN

## Abstract

**Background and Objectives:**

Atherosclerotic changes of arteries are the leading cause for deaths in cardiovascular disease and greatly impair patient’s quality of life. Sphingosine-1-phosphate (S1P) is a signaling sphingolipid that regulates potentially pro-as well as anti-atherogenic processes. Here, we investigate whether serum-S1P concentrations are associated with peripheral artery disease (PAD) and carotid stenosis (CS).

**Methods and Results:**

Serum was sampled from blood donors (controls, N = 174) and from atherosclerotic patients (N = 132) who presented to the hospital with either clinically relevant PAD (N = 102) or CS (N = 30). From all subjects, serum-S1P was measured by mass spectrometry and blood parameters were determined by routine laboratory assays. When compared to controls, atherosclerotic patients before invasive treatment to restore blood flow showed significantly lower serum-S1P levels. This difference cannot be explained by risk factors for atherosclerosis (old age, male gender, hypertension, hypercholesteremia, obesity, diabetes or smoking) or comorbidities (Chronic obstructive pulmonary disease, kidney insufficiency or arrhythmia). Receiver operating characteristic curves suggest that S1P has more power to indicate atherosclerosis (PAD and CS) than high density lipoprotein-cholesterol (HDL-C). In 35 patients, serum-S1P was measured again between one and six months after treatment. In this group, serum-S1P concentrations rose after treatment independent of whether patients had PAD or CS, or whether they underwent open or endovascular surgery. Post-treatment S1P levels were highly associated to platelet numbers measured pre-treatment.

**Conclusions:**

Our study shows that PAD and CS in humans is associated with decreased serum-S1P concentrations and that S1P may possess higher accuracy to indicate these diseases than HDL-C.

## Introduction

Atherosclerosis is a chronic, inflammatory disease that leads to arterial lumen narrowing by the formation of plaques [1–4]. The risk of an acute (thrombo-) embolic event such as stroke or myocardial infarction is associated with plaque vulnerability rather than overall disease stage [4–6]. Today, modern imaging technologies including invasive (intravascular ultrasound) and non-invasive methods (ultrasound, computed tomography, magnetic resonance) can resolve sufficient plaque details to make predictions as to plaque stability [5, 7, 8]. It is therefore important to early identify people at risk so they can benefit from timely pharmacological, endovascular or surgical treatment. There has been a strong focus in research on HDL as a biomarker as well as therapeutical target for atherosclerosis given its strong inverse association with the risk for cardiovascular disease [9]. Other candidate markers include regulators of inflammation and coagulation (recently reviewed in [10]). Sphingosine-1-phosphate is a potent blood-borne signaling compound that is regulating many processes that may promote or attenuate atherosclerosis [11–13]. The effects of S1P in cells are mediated by five cognate S1P receptors, S1PR1-5 [12, 13]. As the S1P/S1PR1 axis is required for the egress of lymphocytes from lymphatic organs into the blood, S1P may regulate inflammatory processes in atherosclerosis [14, 15]. On the other hand, S1P protects endothelial integrity by promoting endothelial barrier function via S1PR1 [16] and, via activation of S1PR3, by stimulating eNOS-derived NO production [17]. As HDL-C is a major S1P carrier in plasma [18], it has been speculated to what extent putative anti-atherogenic effects of HDL-C are due to its S1P content [19]. Two studies have previously investigated whether blood-borne S1P levels are altered in human coronary artery disease (CAD) [20, 21]. While one reported a strong association of serum-S1P with CAD onset and severity [20], the other did not confirm these findings [21]. The reason for this discrepancy is still unclear. Here, we have examined atherosclerotic patients with either peripheral artery disease (PAD) or carotid stenosis (CS) regarding the questions whether their serum-S1P concentrations before surgery differ from those in healthy controls and whether their S1P levels change after surgery.

## Materials and Methods

### Ethics regulations

According to ethics regulations, all samples (controls and patients) and the corresponding blood measurements or medical records were anonymized. Only age and gender was recorded. Patients who agreed to have blood drawn for follow up measurements of S1P (recovery group) signed a separate consent form (Ethics proposal PV3425, approved October 10, 2010).

### Study cohorts

In this single-center study, patients (> 18 years) that presented to the Clinic for Vascular Medicine at the University Heart Center Hamburg-Eppendorf with clinically relevant PAD according to Rutherford classification (stage 3, severe claudication or higher) or CS according to NASCET classification (60% or higher stenosis when symptomatic or 75% stenosis and higher when asymptomatic) were enrolled. Exclusion criteria were severe coronary artery disease, a prevalent coagulation disorder, or a general health score of larger than 3 based on the ASA (American Society of Anesthesiologists) physical status classification system. Blood was drawn at the time of the initial medical examination, serum prepared and stored at -80°C until S1P measurements were performed. At admission to the hospital, patients were asked to consent to blood draws at follow-up examinations for S1P measurements (recovery group). Patients in this group who had received aggressive anti-coagulant treatment with clopidogrel, phenprocoumon or heparin after treatment were retrospectively excluded to avoid confounding effects of these drugs on post-treatment serum-S1P levels. A total of 132 atherosclerotic patients were enrolled, 102 with PAD and 30 with CS as their primary disease; the recovery group consisted of 35 patients. For controls, samples from 174 blood donors at the Institute of Transfusion Medicine, University Medical Center Hamburg-Eppendorf were used to prepare serum, which was then stored at -80°C until S1P measurements were performed. All blood donations were performed in accordance with the latest guidelines of the German federal medical council (Bundesaerztekammer) issued in 2010 that specifically exclude blood donations from subjects with severe health problems including clinically relevant cardiovascular diseases [22].

### S1P measurements

Serum was separated from the clot by centrifugation and stored at -80°C until further processing. S1P measurements were performed using a previously described protocol with minor modifications [23]. Following addition of internal standard (1 nmol/mL C17-S1P, Polar Lipids), serum was de-proteinated by addition of 80% acetonitrile (final concentration: 70%). Extracts were cleared by centrifugation, and subjected to reverse-phase chromatography on a Zorbax SB-C8 column (2.1 x 50 mm, Agilent) at a flow rate of 0.35 mL/min. S1P was eluted by a binary gradient (2.5% methanol, 2.5% acetonitrile, 0.1% formic acid to 30% methanol, 30% acetonitrile, 0.1% formic acid; % = volume %) and measured by a Varian MS 1200 mass spectrometer using multiple reaction mode in which the M+H S1P parent ion (m/z = 380) is fragmented to form a daughter ion at m/z = 264 which is then used for quantitation. The internal standard (C17-S1P) with the m/z 366 to 250 transition was used to correct for variations in sample preparation and instrument response. A calibration curve (0.1–3 nmol/mL S1P) was generated to calculate absolute S1P concentrations in samples.

### Determination of clinical parameters

All measurements of blood parameters (red blood cells (RBCs), white blood cells (WBCs), platelets, hemoglobin, HDL-C, C-reactive protein (CRP) and creatinine) were performed by standardized assays in the Department of Clinical Chemistry/Central Laboratories at the University Hospital Hamburg-Eppendorf (Germany). Medical records were used to identify medications (ASA (acetylsalicylic acid, a.k.a. aspirin), statins, clopidogrel, phenprocoumon (a.k.a. marcumar) and heparin) and the following risk factors and comorbidities: hypertension (RR >140/90 mmHg or higher permanently, or the patient received medication to lower the blood pressure), hypercholesteremia (total cholesterol > 200 mg/dL), obesity (BMI > 30 kg/m^2^), diabetes (chronic hyperglycemia or patient received diabetes medication, oral or s.c.), smoking, COPD (according to the GOLD definition (Global Initiative For Chronic Obstructive Lung Disease)), kidney insufficiency (GFR < 60 mL/min/1.73m^2^) and arrhythmia.

### Statistical analyses

If not stated otherwise, statistical analyses were performed with Prism 6 software (Graph Pad software). Data are presented as median with maximum, minimum and 3^rd^ and 1^st^ quartiles or mean values +/- standard deviation (SD). T-test was used to calculate significance of differences between two groups, the Kruskal-Wallis test was applied when multiple groups were compared. Correlations between S1P and clinical parameters were assessed by calculating the Spearman’s correlation coefficient. To calculate the significance of difference between receiver operating characteristic (ROC) curves, VassarStats software was used (http://vassarstats.net/). The multivariate regression with backward elimination to calculate the difference in serum-S1P between controls and atherosclerotic patients under consideration of all other laboratory parameters was conducted in the Institute of Medical Biometry and Epidemiology using R software [24] More details are given in figure legends.

## Results

### Serum-S1P concentrations are lower in PAD and CS patients compared to healthy blood donors

**Fig 1:**
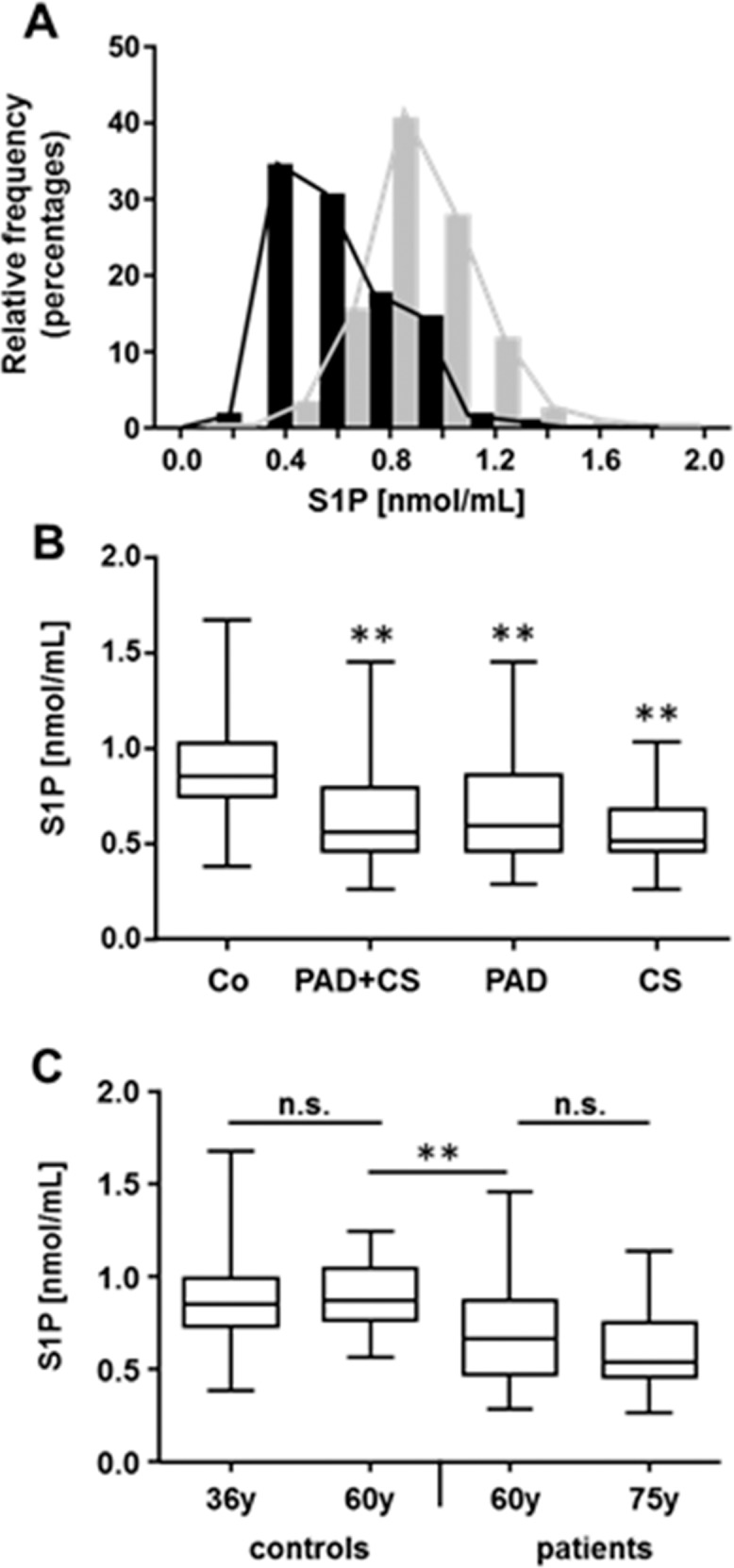
Serum-S1P concentrations in healthy controls and atherosclerotic patients. Serum-S1P was measured in 174 blood donors (controls, Co) and 132 atherosclerotic patients (PAD + CS), 102 with primary PAD and 30 with primary CS. **A**; frequency histograms (percent of subjects vs. serum-S1P) are shown for controls (grey bars) and atherosclerotic patients (black bars). **B**; data are presented as median with maximum, minimum and 3^rd^ and 1^st^ quartiles. Statistical analysis was performed by the non-parametric Kruskal-Wallis test with correction for multiple comparisons. **Indicates significance of difference to controls (P<0.001). **C**; controls and atherosclerotic patients were divided into two groups each to yield one group each with the same median age of 60 years. Four groups with the following median age were formed: Controls (36 years, N = 105 and 60 years, N = 69) and patients (75 years, N = 79 and 60 years, N = 53). Serum-S1P for each group was compared to all other groups using the non-parametric Kruskal-Wallis test with correction for multiple comparisons. ** P<0.001, n.s. = non-significant (P>0.05).

In the control group, serum-S1P concentrations are normally distributed while in atherosclerotic patients, the curve is shifted towards lower S1P concentrations (Fig 1A).

The difference in serum-S1P between controls and atherosclerotic patients is highly significant also when PAD and CS patients are analyzed separately (Fig 1B). Because controls were younger in average compared to PAD or CS patients (Table 1), it is important to determine whether serum-S1P is affected by age. To address this question, age-matched groups of controls and atherosclerotic patients were formed by eliminating the top old patients and the top young controls yielding two groups with a median age of 60 years. The difference in serum-S1P between these now age-matched groups of controls and patients remains highly significant despite smaller group sizes (Fig 1C). Moreover, between the two control groups or the two patient groups of different median age, there is no significant difference in serum-S1P (Fig 1C). Similarly, by analyzing the control group, no gender-specific differences in serum-S1P levels were observed (0.874 +/- 0.223 nmol/mL for men (N = 106) vs. 0.887 +/- 0.194 nmol/mL for women (N = 68)).

**Table 1:** Characteristics of controls and atherosclerotic patient groups.

	Controls	Patients (all)	PAD	CS
**Number enrolled**	174	132	102	30
**Age (years)**	47 (20–71)	70 (41–92)	70 (41–92)	73 (53–84)
**Men (number (%))**	106 (61)	92 (70)	72 (71)	20 (67)

Data for age are presented as median with minimum and maximum. PAD = Peripheral artery disease, CS = carotid stenosis.

To test the possibility that risk factors for atherosclerosis (hypertension, hypercholesteremia, obesity, diabetes and smoking) or comorbidities (COPD, kidney insufficiency, and arrhythmia) affect serum-S1P, medical records were used to divide the atherosclerotic patient cohort into two groups with or without the given condition (Table 2). The S1P levels of the resulting two groups were then compared. None of these tests identified a condition that significantly influences serum-S1P concentrations in atherosclerotic patients (Table 2).

**Table 2:** Association of serum-S1P levels with risk factors for atherosclerosis and comorbidities in atherosclerotic patients.

	Frequency (percentage)	S1P^(1)^ without mean +/- SD	S1P^(1)^ with mean +/- SD	significant (P<0.05)
**Hypertension**	30	0.730 +/- 0.265	0.670 +/- 0.265	no
**Hypercholesteremia**	42	0.716 +/- 0.268	0.647 +/- 0.261	no
**Obesity**	7	0.696 +/- 0.265	0.573 +/- 0.268	no
**Diabetes**	27	0.688 +/- 0.268	0.685 +/- 0.264	no
**Smoking**	55	0.695 +/- 0.220	0.681 +/- 0.299	no
**COPD**	8	0.682 +/- 0.024	0.742 +/- 0.072	no
**Kidney insufficiency**	8	0.691 +/- 0.268	0.642 +/- 0.245	no
**Arrhythmia**	11	0.700 +/- 0.268	0.590 +/- 0.227	no

For every risk factor or concomitant disease analyzed, medical records were used to classify patients (N = 132) into two groups (without and with the condition). For each group, serum-S1P concentrations pre-treatment are shown (mean +/- SD). Significance of differences between patients with or without the respective condition was tested by two-tailed T-test.

^(1)^S1P concentrations are [nmol/mL]; COPD = Chronic obstructive pulmonary disease.

As all blood parameters measured in controls and atherosclerotic patients differed significantly between the two groups (Table 3), a multivariate regression analysis was performed to control for the potential influence of these parameters on serum-S1P. By stepwise eliminating the variable with the highest P-value, a final model was obtained that shows a significant effect of hematocrit and platelet numbers (Table 4). Under consideration of these variables, atherosclerotic patients have 0.183 nmol/mL less serum-S1P compared to controls (Table 4).

**Table 3:** Association of blood parameters with serum-S1P in controls and atherosclerotic patients pre-treatment.

	Controls	Patients	P
**S1P [nmol/mL]**	0.879 +/- 0.212	0.687 +/- 0.266	<0.0001
**RBCs/pL**	4.84 +/- 0.41	4.41 +/- 0.53	<0.0001
**WBCs/nL**	6.40 +/- 1.62	8.19 +/- 2.14	<0.0001
**Platelets/nL**	234 +/- 56	271 +/- 100	<0.0001
**Hb [mg/dL]**	14.5 +/- 0.1	13.4 +/- 1.8	<0.0001
**HCT [%]**	43.6 +/- 3.1	40.2 +/- 5.0	<0.0001
**HDL-C [mg/dL]**	62.7 +/- 18.2	51.5 +/- 19.8	<0.0001
**Creatinine [mg/dL]**	0.94 +/- 0.17	1.20 +/- 1.14	<0.005

Data are presented as mean +/- SD (N = 174 for controls and N = 132 for patients except for HDL-C (N = 127)). Significance (P) between controls and patients was calculated by two-tailed T-test. RBCs = red blood cells, WBCs = white blood cells, Hb = hemoglobin, HCT = hematocrit, HDL-C = high density lipoprotein-cholesterol

**Table 4:** Multivariate regression analysis with backward elimination.

	Regression- Coefficient	Confidence Interval (95%)	P
**vascular patient**	-0.18302	-0.24021–-0.12583	<0.001
**HCT**	0.00998	0.00354–0.01641	0.003
**platelet numbers**	0.00068	0.00034–0.00100	<0.001

This analysis was performed to calculate the difference of serum-S1P between controls and atherosclerotic patients while controlling for blood parameters that significantly influence S1P levels. Calculations were performed using R-software [24], HCT = hematocrit

**Fig 2:**
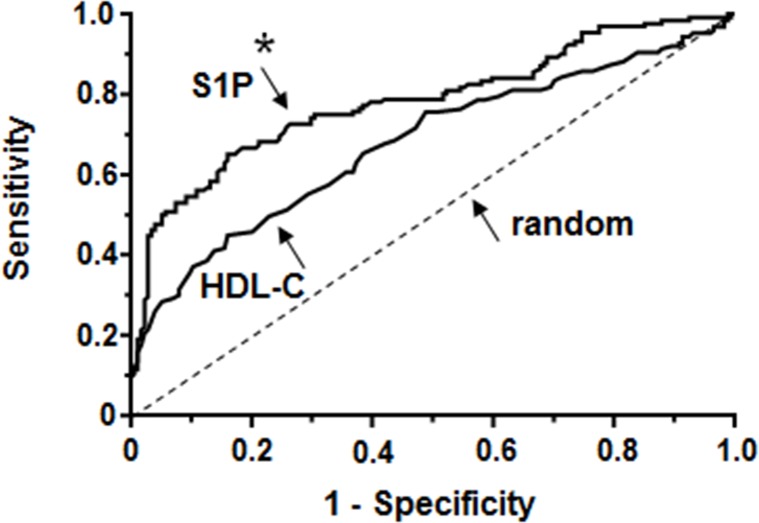
S1P may have more power to indicate atherosclerosis (PAD and CS) than HDL-C. Receiver operating characteristic (ROC) curves were generated for S1P and HDL-C measured in controls (N = 174) and atherosclerotic patients (N = 132). The dotted line represents the line of identity. *the AUC for S1P and HDL-C are significantly different (P <0.05)

As low levels of HDL-C have long been associated with an increased risk of cardiovascular diseases, ROC curves were generated to compare the potential of HDL-C with that of S1P to indicate atherosclerosis (PAD and CS; Fig 2). From this analysis, both compounds possess highly significant potential to indicate these diseases (P<0.0001) whereby S1P had a larger area under the curve (AUC = 0.78, 95% confidence interval (CI) 0.73–0.84) than HDL-C (AUC = 0.68, 95% CI 0.62–0.74) (P<0.05).

### Serum-S1P levels rise after treatment

From a total of the 132 atherosclerotic patients, 35 patients agreed prospectively to have blood drawn for S1P measurements during follow-up examinations that occurred between one and six months after treatment (recovery group, Table 5). Patients who underwent aggressive anti-coagulant therapy with clopidogrel, phenprocoumon or heparin after surgery were not considered because of potentially confounding effects of these drugs on serum-S1P levels. It should be noted that almost every patient took ASA or a statin before and after surgery (Table 5).

**Table 5:** Characteristics of the recovery group.

	Recovery group
**Number enrolled**	35
**PAD / CS**	27 / 8
**Age (years)**^(1)^	69 (47–81)
**Men (%)**	69
**Medication before treatment**^(2)^	
** ASA (%)**	94
** Statins (%)**	94
**Medication after treatment**^(3)^	
** ASA (%)**	100
** Statins (%)**	97
**Treatment: open / endovascular**	28 / 7

^(1)^Data are presented as median with minimum and maximum.

^(2)^Percentages given are based on information of 16 patients.

^(3)^Percentages given are based on information of all 35 patients.

**Fig 3:**
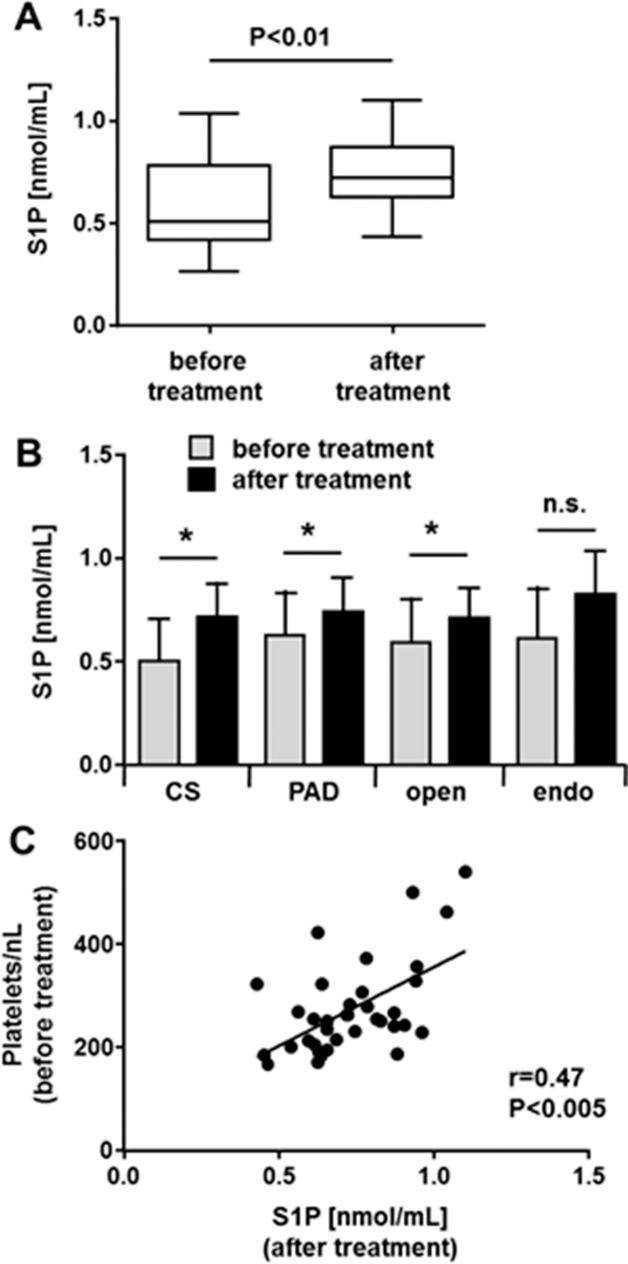
Serum-S1P levels rise after treatment. Serum-S1P was measured in 35 patients between one and six months after treatment (recovery cohort). **A**; serum S1P in the recovery cohort (N = 35) was compared before and after treatment to restore blood flow. Data are presented as median with maximum, minimum and 3^rd^ and 1^st^ quartiles. Statistical analysis was performed by two-tailed, paired T-test. **B**; the recovery cohort was classified into two groups at a time, CS patients (N = 8) and PAD patients (N = 27) or as to the form of treatment (open (N = 28) or endovascular (N = 7)). The serum-S1P concentrations (mean +/- SD) before and after treatment are shown for each group. Statistical analysis was performed by two-tailed T-test. *P<0.05; n.s. = non-significant. **C**; using data obtained from the recovery cohort, a regression analysis was performed for platelet numbers pre-treatment and serum-S1P in recovery (r = Spearman correlation coefficient).

Regarding age or gender, there was no significant difference between the recovery group and the entire patient cohort (Tables 1 and 5). All 8 CS patients in the recovery group were treated by thromboendarterectomy (TEA), 20 from 27 PAD patients (74%) underwent open surgery (TEA and/or bypass), the others (26%) endovascular surgery (percutaneous transluminal angioplasty and/or stenting). When compared to pre-treatment S1P levels, S1P concentrations after treatment were significantly increased (Fig 3A). This increase is independent of whether patients suffered PAD or CS, or whether patients were treated by open or endovascular surgery (Fig 3B). To identify parameters that may regulate S1P levels in recovery, correlations between S1P concentrations and blood parameters measured pre-treatment (see Table 3) were analyzed. The only significant correlation found was for platelet numbers (Fig 3C).

## Discussion

The major findings of this study are that serum-S1P concentrations in patients with PAD or CS at their time of admission are significant lower when compared to healthy controls and that S1P levels rise after treatment. Notably, serum-S1P levels pre-treatment may have more power to indicate atherosclerosis (PAD or CS) than HDL-C.

### Serum-S1P concentrations in controls

Our control group of blood donors can be considered healthy as all are medically screened and subjects with obvious health problems are legally excluded from donations. In controls, we found that serum-S1P concentrations range from 0.4 nmol/mL to 1.7 nmol/mL and values are normally distributed (Fig 1). To our knowledge, there are no reports of serum-S1P levels in similarly sized control cohorts to compare our data with. Two previous studies report average serum-S1P levels in controls of 0.484 and 0.634 nmol/mL but group size with 8 and 11 individuals, respectively, was very small [20, 25]. Absolute mean numbers of different studies are probably also difficult to compare to each other when different measuring technologies are used. In a larger control cohort of 85 individuals, measurements of plasma-S1P showed a similar distribution of S1P levels to our study with an approximately 4-fold difference between minimum and maximum values [21]. While relative differences in serum- or plasma S1P concentrations between groups can be compared across different studies, for comparisons of absolute numbers, a standardized assay may need to be developed. The question as to measure S1P in serum or plasma needs to be addressed as well. The difference in S1P concentrations between serum and plasma is mainly due to platelets which release S1P during coagulation [26, 27]. For the present study, we have chosen to use serum hoping for less variability in S1P numbers as platelets may leak S1P during storage time of the blood sample, which may differ significantly between individual samples. Because our control cohort was younger in age and had a higher percentage of women than our patient cohort, we needed to investigate whether serum-S1P is influenced by age and gender. This does not seem to be the case (see text in the results section and Fig 1C). It should also be noted that blood samples of patients and controls were handled the same way and that almost all blood samples were processed within 24 hours. Only when blood was drawn exceptionally on weekends, samples were possibly kept 36–48 hours at 4°C before centrifuged. We have previously taken two blood samples from the same individual (N = 3), centrifuged one 30–60 min after drawing and the other after 2 days at 4°C. No blood storage time-dependent differences in serum-S1P concentrations were found; also, at -80°C, S1P is stable in serum at least for several months (unpublished observations). At this point we rule out that the differences in serum-S1P found in controls and atherosclerotic patients are due to differences between the two cohorts regarding sample processing or storage, age or gender.

### Low serum-S1P concentrations are associated with PAD and CS

Based on many potential pro- or atherogenic effects of S1P on the vasculature, the main objective of this study was to determine whether serum-S1P levels are altered in atherosclerosis and whether they change after treatment to restore blood flow. Compared to controls, patients with PAD or CS have significant lower serum-S1P levels before they undergo invasive treatment to restore blood flow (Fig 1). None of the risk factors for atherosclerosis nor any of the comorbidities analyzed were found to significantly influence serum-S1P levels (Table 2). As all blood parameters measured differed between controls and atherosclerotic patients (Table 3), a multivariate regression analysis with backward elimination was performed to determine their influence onto serum-S1P. The final model shows that hematocrit and platelet numbers positively affect serum S1P (Table 4). Under consideration of the effect of hematocrit and platelet numbers, atherosclerotic patients have 0.183 nmol/mL less S1P than controls. It should be noted that without controlling for hematocrit and platelet numbers, this difference is slightly higher (-0.192 nmol/mL; calculated from Table 1). As platelets are positively associated with serum-S1P but atherosclerotic patients have lower serum-S1P and higher platelet numbers compared to controls (Table 3), hematocrit appears to be the major confounding factor. The positive association between hematocrit and serum-S1P confirms that erythrocytes are a major source of plasma S1P [28, 29].

An interesting observation is that HDL-C concentrations are not correlated to serum-S1P levels, although HDL-C is an important carrier for S1P in plasma [18]. Thus, the low HDL-C levels in patients are probably not directly linked to their decreased S1P levels. At present, mechanisms underlying the decrease in serum-S1P concentrations in atherosclerotic patients are speculative. Possibilities include medications, particularly ASA and statins that are taken by almost every patient pre-treatment (Table 1) and in certainly less frequency by controls. Indeed, ASA has recently been shown to block the thrombin-receptor PAR-1 (protease-activated receptor-1)-stimulated release of S1P in platelets *ex vivo* [30]. Our own finding, however, that serum-S1P levels rise after treatment despite almost all patients take ASA, argues against a strong negative effect of ASA on serum-S1P concentrations. In contrary, medication of patients with mild to moderate atherosclerosis with rosuvastatin has been reported to cause an increase of HDL-C as well as plasma-S1P, though the increase in S1P did not reach statistical significance [31]. Such an effect, however, would result in an elevation of serum-S1P levels in patients. Taken together, we do not believe that ASA or statins are major determinants to regulate serum-S1P in atherosclerotic patients. Notably, other patient cohorts exhibiting decreased serum- or plasma S1P levels have recently been described, subjects with acute dengue infection [32] and sepsis [33–35]. Both diseases are characterized by increased vascular permeability. As endothelial barrier function depends on S1P receptor-1 signaling, the authors suggest the possibility that low S1P levels contributes to this complication. Common between dengue fever, sepsis and atherosclerosis is endothelial damage and the endothelium may be an important source for serum-S1P [36]. Although data from bone marrow transplant experiments between wild-type and inducible sphingosine-kinase knock-out mice indicate a critical role for hematopoietic cells to define plasma-S1P levels [29], a role for the endothelium is supported by observations that mice that have been made thrombocytopenic, anemic or leukopenic all showed normal plasma S1P levels [36]. An interesting possibility is therefore that the decrease of serum-S1P levels in atherosclerotic patients is due to endothelial dysfunction which would make S1P a marker for endothelial integrity. Measurements of endothelial function by FMD (Flow-mediated dilatation) have been shown to predict carotid intima-media thickness [37] and it would be worth investigating whether FMD measurements are associated with serum-S1P concentrations.

### Serum-S1P may have a prognostic value for atherosclerosis (PAD and CS)

As atherosclerosis develops well before any clinical symptoms become obvious, a marker to predict the progression of atherosclerosis would be of tremendous value. The strongest association between atherosclerosis and a blood-borne parameter so far exists for HDL-C which spurred intensive research, also to use elevation of HDL-C levels as a therapeutic treatment; the results of these efforts, however, raised doubts if HDL-C concentrations are directly linked to atherosclerosis [38, 39]. More successful approaches may lie in targeting specific HDL functions such as the extraction of cholesterol from macrophages [9]. After S1P has been identified as the component in HDL-C that stimulates NO production by the endothelium, it was proposed that beneficial effects of HDL are actually due to its S1P content [19]. Given this relationship between S1P and HDL-C, we sought to compare their potential to indicate atherosclerosis (PAD and CS) by generating ROC curves and calculating the AUC. In our analysis, S1P emerged with a higher AUC than HDL-C (Fig 2). Even considering that the data set for serum-S1P derived from atherosclerotic patient cohort is very small compared to the vast data sets available for HDL, this finding suggests that S1P may be a potent biomarker to indicate PAD and CS and may also, at least in part, regulate these diseases independently of HDL-C concentrations.

### Serum-S1P levels rise after treatment

Follow-up measurements of serum-S1P showed that S1P levels rise after treatment (Fig 3A). This increase is independent of the primary atherosclerotic disease (PAD or CS) and of whether patients underwent open or endovascular surgery (Fig 3B). As almost all patients took ASA and a statin before and after therapy and because we excluded patients with any other pharmaceutical anti-coagulation therapy, we rule out the possibility that this increase of serum-S1P during recovery is due to drugs. It is an intriguing possibility that this increase of S1P supports healing by stimulating endothelial survival and proliferation. The observation that platelet numbers (pre-treatment) correlate with serum-S1P levels after treatment (Fig 3C) may suggest that mainly platelets are responsible for this increase in serum-S1P during recovery. Platelets are thought to be a major contributor to plasma S1P as they store high amounts of S1P that they release upon stimulation [26, 27] and an association between serum-S1P and platelet numbers has been previously observed [27, 32]. Platelet-dependent release of S1P may also contribute to the different results of the two reports aforementioned that studied associations between blood-borne S1P and coronary artery disease [20, 21].The positive correlation with coronary stenosis and serum-S1P observed in one report [20] may be caused by a high number of patients with acute thrombotic events, where platelets then generate S1P. In contrast, the cohort of the other study [21] may have had a lower frequency of patients with acute thrombotic events and therefore, the authors made the same observation we did in our atherosclerosis patients that is that serum-S1P levels in patients are lower than in healthy controls.

### Limitations

We see the major limitation of this study in the rather loose definition of the atherosclerotic patient cohort. A patient admitted with clinically relevant PAD may as well have diseased carotid arteries, or the other way around. Moreover, patients may suffer atherosclerotic changes at other sites in addition, and it cannot be ruled out that changes in serum-S1P depend on the localization of plaques. It is also unknown whether changes in serum-S1P are associated with disease stage or plaque burden. Clearly, larger cohorts with better defined plaque morphologies, plaque burden and disease localizations need to studied to further investigate a clinical role for S1P in human atherosclerosis.
